# Characterization of *Escherichia coli* isolated from urinary tract infection and association between virulence expression and antimicrobial susceptibility

**DOI:** 10.1186/s12866-022-02506-0

**Published:** 2022-04-06

**Authors:** Safoura Derakhshan, Sanaz Ahmadi, Erfan Ahmadi, Sherko Nasseri, Abbas Aghaei

**Affiliations:** 1 Lung Diseases and Allergy Research Center, Research Institute for Health Development, Kurdistan University of Medical Sciences, Sanandaj, Iran; 2 Department of Microbiology, Faculty of Medicine, Kurdistan University of Medical Sciences, Sanandaj, Iran; 3 Social Determinants of Health Research Center, Research Institute for Health Development, Kurdistan University of Medical Sciences, Sanandaj, Iran; 4 Student Research Committee, Kurdistan University of Medical Sciences, Sanandaj, Iran; 5 Cellular and Molecular Research Center, Research Institute for Health Development, Kurdistan University of Medical Sciences, Sanandaj, Iran

**Keywords:** *Escherichia coli*, Gene expression, Hemolysin, Minimum inhibitory concentration, Pathogenicity

## Abstract

**Background:**

The capacity of antibiotics to modulate bacterial virulence has raised concerns over the appropriateness of antibiotic therapies, including when dosing strategies fall below sub-therapeutic levels. In this work, we investigated the ability of antibiotics to influence virulence in *Escherichia coli* isolated from urinary tract infection (UTI).

**Results:**

Out of 120 isolates, 32.5% carried *pap*, 21.7% carried *hlyA*, and 17.5% carried *cnf*. The predominant B2 phylogroup was significantly associated with the quinolone-resistant isolates. A significant association was seen between the presence of *hlyA* hemolysin and susceptibility to ceftriaxone and ciprofloxacin (*P* < 0.05). Sub-inhibitory concentrations of both antibiotics reduced the levels of *hlyA* expression and hemolysis in isolates treated with antibiotics compared to untreated isolates (*P* < 0.05). Growth rate assay showed that the decrease in *hlyA* expression was not an effect of decreased growth rate.

**Conclusion:**

Our study indicated the inhibitory effect of ciprofloxacin and ceftriaxone on the level of hemolysis, suggesting that the sub-inhibitory concentrations of these antibiotics may affect the outcome of infections. Further studies, including animal models may elucidate the outcome of virulence modulation by these antibiotics in UTI pathogenesis.

## Background

Antibiotics still remain the mainstay treatment for bacterial infections; however, inappropriate use of antibiotics may promote a rapid emergence of resistant strains and give bacteria an opportunity to modulate their own pathogenicity. When in lethal levels, antibiotics kill or inhibit growth of bacteria; they also may act as signal molecules at their sub-minimal inhibitory concentrations (sub-MICs) [1].

Urinary tract infections (UTIs) are among the most common infections encountered in many countries and *Escherichia coli* is by far the most frequent cause of UTIs [2]. *E. coli* strains are classified into several major phylogenetic groups (phylogroups) [3]. Extraintestinal pathogenic *E. coli* strains are mainly derived from phylogroups B2 or D, and commensal isolates fall into groups A or B1 [4]. Strains of uropathogenic *E. coli* (UPEC) are characterized by their ability to produce distinctive virulence factors such as adhesins, toxins, and siderophores. Several uropathogenic virulence genes are located in large contiguous blocks known as pathogenicity islands (PAIs) and through this they can be transmitted to other bacteria by horizontal gene transfer. Cytotoxic necrotizing factor type 1 (*cnf-1* gene), hemolysin (*hlyA* gene), and pyelonephritis- associated pilus (*pap* gene), play important roles in the pathogenicity of UPEC strains. The expression of adhesion organelles such as P pili enables UPEC to bind and invade host cells. Binding of this adhesin to the renal tissue may lead to mucosal inflammation and pain associated with UTIs. Toxins are also important virulence factors in the pathogenicity of UPEC. The CNF1 has been shown to facilitate dissemination of strains in the urinary tract. This toxin causes bladder cell exfoliation and enhances bacterial access to the underlying tissues [5, 6]. HlyA, which is encoded by the *hlyCABD* operon, is the most important secreted virulence factor of UPEC. This toxin provides UPEC with the ability to cause tissue damage, cross mucosal barriers, release host nutrients and damage immune cells. Initially hemolytic *E. coli* was separated into two, somewhat confusing categories; however, it was later found that the hemolysins are encoded by a set of homologous genes and all *hlyCABD*-encoded activities were designated as the *E. coli* hemolysin [7]. A novel pore-forming chromosomal hemolysin not related to HlyA, cytolysin A (ClyA, also referred to as HlyE or SheA), was first identified in *E. coli* K12. The ClyA protein is not produced at phenotypically detectable levels under standard conditions. Furthermore, the incidence of functional alleles of *clyA* showed a correlation with intestinal *E. coli* pathotypes, and all *E. coli* strains isolated from extraintestinal infections carried no *clyA* or only nonfunctional *clyA*. Therefore, hemolytic ability of UPEC is linked to the production of HlyA [8].

In recent years, UTIs have become increasingly difficult to treat due to the widespread emergence of resistance to antibiotics, especially to newer broad-spectrum agents, such as fluoroquinolones, which are DNA-damaging antibiotics and cephalosporins, which promote defective cell wall synthesis through specific interactions with penicillin binding proteins (PBPs) [9]. Exposure to multi-drug resistant *E. coli* may lead to hard to treat diseases. The situation becomes much more complicated when the antibiotic is incorrectly used. Although in clinical therapeutics antibiotics are usually used in high doses, sub-MICs of agents may occur. When the antibiotic reaches a sub-inhibitory concentration, it gives bacteria an opportunity to become resistant and to modulate their own virulence. Modulation of gene expression during antibiotic treatment could alter the course of bacterial pathogenesis [1]. Understanding how UPEC may respond to non-lethal concentrations of antibiotics is important, because successful treatment of UTI depends on the antibiotic treatment, and maintenance of lethal concentrations of antibiotics during the treatment is not always possible [10]. For these reasons, studying the effect of sub-MIC antibiotics on the expression of bacterial virulence factors may provide more information for proper use of antimicrobial agents.

In this study, we investigated antibiotics for their capacity to influence virulence traits in *E. coli* isolated from UTI. The presence of *hlyA*, *cnf1* and *pap* was investigated, whose products are instrumental in the pathogenesis of UTIs. These genes are physically linked in PAI domains, which are prevalent in UTI isolates [9].

## Materials and methods

### Bacterial isolates and antimicrobial susceptibility testing

A total of 120 non-duplicate *E. coli* isolates were obtained from urinary specimens of UTI patients who were referred to the hospitals affiliated to Kurdistan University of Medical Sciences in Sanandaj, Iran. Sanandaj is the capital of Kurdistan Province in western Iran and patients were referred to its hospitals from all parts of the province. UTI was defined according to the guidelines of European Association of Urology [11]. Identification of *E. coli* was done by the standard tests including Gram staining, citrate utilization, lactose fermentation, motility, ability to produce indole, and lysine decarboxylation [12]. Dissimilarities between the isolates were assessed using repetitive extragenic palindromic-polymerase chain reaction (REP-PCR) method [13]. The isolates were preserved at -70 °C in Trypticase soy broth (Q-lab, Canada), containing 15% v/v glycerol for further investigations.

Antimicrobial susceptibility was determined via the disk diffusion method on Mueller–Hinton agar plates (Q-lab, Canada) according to the guidelines described by Clinical and Laboratory Standards Institute (CLSI) 2020 [14]. The isolates were tested against 14 antimicrobials (Rosco, Denmark): ceftriaxone (Cax, 30 μg), ceftazidime (Caz, 30 μg), imipenem (Imp, 10 μg), aztreonam (Azt, 30 μg), cefepime (Cpe, 30 μg), cefoxitin (Cfx, 30 μg), ciprofloxacin (CIP, 5 μg), norfloxacin (NX, 10 μg), nalidixic acid (NA, 30 μg), gentamicin (GM, 10 μg), amikacin (AK, 30 μg), tetracycline (TE, 30 μg), trimethoprim-sulfamethoxazole (TS, 1.25/23.75 μg), and nitrofurantoin (NI, 300 μg). Bacterial isolates were classified as sensitive (S), intermediately sensitive (I), or resistant (R) to the antibiotics. *E. coli* ATCC 25,922 was used as quality control strain.

### Detection of virulence genes and phylogenetic groups

Genomic DNA was extracted from the isolates by boiling method. Briefly, the overnight culture suspensions of isolates were centrifuged and the pellets were suspended in 200 µl sterile deionized water. The suspensions were then heated at 100 °C for 10 min and immediately placed on ice for 5 min. Samples were centrifuged and after quantitative and qualitative evaluation, the supernatants were stored at -20 °C as DNA template.

The extracted DNA was used for identification of the virulence genes (*pap*, *cnf1*, and *hlyA*) by PCR. Primers (Table 1) were either previously published or designed using the Primer3 online tool (https://primer3.ut.ee). PCR reactions were performed in a 25 μL reaction mixture containing 12.5 μL of 2X Taq PCR Master Mix (SinaClon, Iran: 0.08 U of Taq polymerase/μL, 0.4 mM of each dNTP, and 3 mM MgCl_2_), 0.4 µM of each primer, and 3 µL DNA extract. The PCR reactions were carried out on a thermal cycler (Eppendorf, Germany) under the following conditions: initial denaturation of 5 min at 94 °C, followed by 30 cycles of denaturation of 1 min at 94 °C, annealing of 1 min at different temperatures (Table 1), extension of 1 min at 72 °C, and a final extension of 5 min at 72 °C.

**Table 1:** Genes, putative function of products, primers, annealing temperatures and predicted size of amplification products

Gene	Product	Primer sequence (5′-3′)	Product size (bp)	Annealing temperature (°C)	Reference
*pap*	P-fimbriae	GACGGCTGTACTGCAGGGTGTGGCG/ ATATCCTTTCTGCAGGGATGCAATA	328	65	[15]
*hlyA*	Hemolysin A	TTGAGTCACACCTGGGAGAC/ CCGTGTAATTACCCGCTTCG	162	57	This study
*cnf1*	Cytotoxic necrotizing factor 1	TGCGGGTGTAAATTCAGTGC/ TCTCGTTGAGCCTCACTGTT	186	56	This study
*hlyE*	Hemolysin E	GAAACCGCAGATGGAGCATT/ CGCAACACCACACCATTCAT	219	56	This study
16S rRNA	Ribosomal RNA	GAATGCCACGGTGAATACGTT/ ACCCACTCCCATGGTGTGA	64	57	[16]

The distribution of phylogenetic groups among the isolates was determined as described by Clermont et al., and phylogroups were assigned based on the presence or absence of bands according to the previously defined criteria [3]. The amplified products were separated on a 1.5% agarose gel (SinaClon) in Tris–Borate EDTA (TBE) buffer and visualized under UV after staining with DNA Safe Stain (SinaClon). A molecular weight standard (50 bp ladder, SinaClon) was included on each gel.

### Determination of MIC and evaluation of antibiotic effects on gene expression

Antibiotic powders used in the gene expression analysis were provided from Sigma (Germany). MIC were determined by broth dilution method in Mueller–Hinton broth (MHB, Merck, Germany) supplemented with calcium (25 mg/L) and magnesium (12.5 mg/L) (cation adjusted MHB, CAMHB) according to the CLSI guidelines [17].

Briefly, two-fold serial dilutions of antibiotics in CAMHB were prepared. A turbidity of 0.5 McFarland standard (~ 1 × 10^8^ colony forming unit (cfu)/mL) was prepared from overnight cultures of the isolates. The prepared suspensions were diluted to ~ 1 × 10^6^ cfu/mL and inoculated into CAMHB containing varying concentrations of the antibiotic to prepare a concentration of 5 × 10^5^ cfu/mL. Cultures were grown for 16–20 h at 35 °C and MIC was determined as the lowest concentration of an antimicrobial agent that prevents visible growth of the isolates. Cultures without the antibiotics and without bacteria were used as growth and negative control, respectively. *E. coli* ATCC 25,922 was used as control strain.

### RNA extraction, cDNA synthesis and real time-PCR (RT-PCR) assay

Total RNAs were isolated by the RNX-Plus kit (SinaClon), according to the manufacturer’s instructions. Sub-MICs (0.25 MIC) of antibiotics and cultures without the antibiotics (growth control) were subjected for RNA extraction by RT-PCR.

Briefly, the aliquots of each culture were centrifuged at 10,000 rpm for 2 min. The pellets were washed and suspended in 1 mL ice cold RNX-PLUS solution (containing guanidine and phenol) with vigorous vortexing. Then, chloroform was added and the samples were incubated with periodical mixing for 5 min at 4 °C and centrifuged at 12,000 rpm for 15 min at 4 °C. The aqueous phase was transferred to a fresh RNase-free microtube and mixed with equal volume of isopropanol to precipitate RNA. After gently mixing and incubation on ice for 15 min, the samples were centrifuged at 12,000 rpm for 15 min at 4 °C. The supernatants were discarded and the RNA pellets were washed with 1 mL 75% ethanol to remove isopropanol and then centrifuged at 7500 rpm for 8 min at 4 °C. This step was repeated twice. The air-dried RNA samples were suspended in 50 μL of nuclease-free water.

RNA integrity was verified by analyzing 2 μL of the total RNA by 1.5% agarose gel electrophoresis in TBE buffer. RNA concentration and purity were determined by measuring the absorbance at 260 nm (A260) and by calculating the ratio of A260/A280 using NanoDrop spectrophotometer (Bio-Tek, Canada). High quality (A260/280 = 1.8–2.0) of total RNAs was obtained in the extraction assays.

cDNA was synthesized using the Easy cDNA Synthesis kit (Parstous, Iran) as recommended by the manufacturer with 100 ng of total RNA as the template. RNA was mixed with 10µL buffer mix (2X), 2µL enzyme mix and nuclease-free water in a reaction volume of 20 μL. The mixture was incubated at 25 °C for 10 min and then incubated at 47 °C for 60 min. The reaction was stopped by 5 min incubation in 85 °C.

The resulting cDNA was used as a template for RT-PCR amplification. The housekeeping gene used for normalization was 16S rRNA gene (Table 1). The RT-PCR reactions were conducted in a reaction volume of 20 μL containing 10 μL of 2X SYBR Green RT-PCR mix (which included HotStarTaq DNA Polymerase, PCR buffer, MgCl2, dNTP mix and dye; SinaClon), 0.4 µM of each primer, and cDNA amount corresponding to 100 ng RNA. Water was included as non template control. Reactions were run using the Rotor-Gene 6000 version 7.1 (Corbett Research, Australia), analyzed using the system’s software and compared to generate fold changes. A standard curve was established for assessing the reaction efficiency. The RT-PCR assay was optimized to the initial activation step of 95 °C for 7 min, followed by 40 cycles of denaturation at 95 °C for 15 s, annealing at 58 °C of 20 s for all the studied genes, and extension at 72 °C for 20 s. Melting curve analysis was carried out in order to analyze the accuracy of experiments. The results were analyzed using the comparative Ct method (2^−ΔΔCT^) method, according to the formula developed by Schmittgen and Livak [18].

### Growth rate analysis

Growth rate assay was performed as previously described with minor modifications [19]. Briefly, a suspension with the turbidity equivalent to the 0.5 McFarland standard (~ 1 × 10^8^ cfu/mL) was prepared from overnight culture of each isolate and adjusted to 5 × 10^5^ cfu/mL in CAMHB medium without and with antibiotics (0.25 MIC). The cultures were incubated for 16 h at 35 °C with shaking at 200 rpm and bacterial growth was monitored by measuring the optical density of the culture at 625 nm using a plate reader (Bio-Tek, Canada).

### Hemolysis assay

Hemolytic activity was assessed by hemolysis of sheep red blood cells (RBCs), as described previously [20]. The 0.5 McFarland suspensions (~ 1 × 10^8^ cfu/mL) prepared from overnight culture of isolates were diluted to 5 × 10^5^ cfu/mL in CAMHB medium without and with 0.25 MIC of antibiotics and incubated for 16 h at 35 °C. Bacterial samples (0.5 mL) were taken and the cells were pelleted by centrifugation (10,000 rpm, 5 min) and filter sterilization. The supernatants were removed and 0.2 mL of each supernatant was mixed with an equal volume of RBC suspensions (4% in normal saline) (Bahar Afshan, Iran). After incubation at 37 °C for 60 min, the cells were pelleted by centrifugation at 10,000 rpm for 5 min and 0.1 mL of each supernatant was transferred into an untreated flat-bottom 96-well microtiter plate (Jet Biofil, China). The optical densities of the supernatants were determined at 450 nm using a plate reader (Bio-Tek, Canada). RBCs mixed with sterile deionized water were used as a positive control, whereas RBCs with normal saline or CAMHB medium were served as negative controls.

The percentage of hemolysis was calculated by comparison with the untreated drug free culture. Sterile culture medium was designated as the standard for 0% hemolysis and a drug free culture supernatant was served as the standard for 100% hemolysis. The hemolysis percentage was calculated as follows: absorbance of the sample-absorbance of the negative control/absorbance of the untreated culture-absorbance of the negative control × 100 [21].

To ensure that hemolytic activity in our isolates was attributed to the presence of *hlyA*, the presence of *hlyE* was investigated by PCR (Table 1)**.** The conditions were: initial denaturation of 5 min at 94 °C, followed by 30 cycles of denaturation of 1 min at 94 °C, annealing of 1 min at 56 °C, extension of 1 min at 72 °C, and a final extension of 5 min at 72 °C. The products were separated on a 1.5% agarose gel in TBE buffer and visualized under UV after staining with DNA Safe Stain.

### Statistical analysis

All experiments were performed in triplicate and the results were expressed as the mean of three independent experiments. SPSS Version 16 (IBM SPSS Statistics, USA) was used for statistical analysis. Descriptive statistics, Chi-square or Fisher's exact test were used to evaluate the relationship between the variables. In RT-PCR, the values obtained for each antibiotic were compared to those obtained for the control (without antibiotic) by the paired t test [22]. A *P* < 0.05 was considered significant.

## Results

### Characteristics of patients

A total of 120 epidemiologically unrelated *E. coli* isolates were analyzed from patients with UTI. The age range of patients undergoing survey was from 1 to 83 years old with the average of approximately 34.9 years. Seventy six (63.3%) of the 120 isolates came from the outpatients and 44 (36.7%) were isolated from the inpatients, which were hospitalized in the pediatric ward (*n* = 19 patients), women specialized ward (*n* = 12), internal medicine (*n* = 10), emergency (*n* = 7), and other wards (*n* = 6). There were 97 females (80.8%) and 23 males (19.2%).

### Antimicrobial susceptibility and phylogenetic analysis

Susceptibility results showed that 96.7% of the isolates were susceptible to NI (*n* = 116/120), 91.7% to Cfx (*n* = 110), and 87.5% to Imp (*n* = 105). They were excluded from statistical analysis of difference among groups. The sensitivity to other antibiotics was: 74.2% (*n* = 89) to AK, 64.2% (*n* = 77) to Caz, 61.7% (*n* = 74) to each NX and Azt, 55% (*n* = 66) to Cax, 51.7% (*n* = 62) to GM, 45.8% (*n* = 55) to Cpe, 44.2% (*n* = 53) to NA, and 40.8% (*n* = 49) to TE. The least effective antibiotics were CIP (33.3% susceptibility, *n* = 40), and TS (28.3% susceptibility, *n* = 34).

**Fig. 1:**
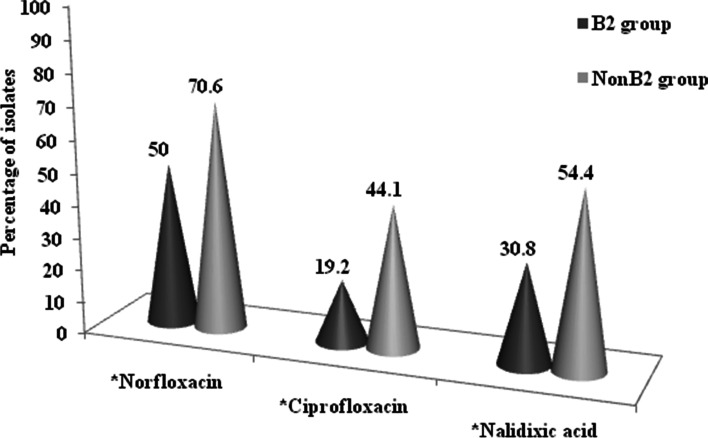
Prevalence of susceptibility to antibiotics in B2 and nonB2 group. Only significant associations are shown. *: *P* < 0.05

The predominant phylogenetic group among the 120 isolates was B2 (*n* = 52, 43.3%), followed by E (*n* = 10, 8.3%), and clade I (*n* = 8, 6.7%), respectively. Groups A, F, C, and D were each represented by 4 isolates (3.3%). Thirty four isolates (28.3%) were classified as unknown. A comparison between the B2 and non-B2 phylogroups showed that the B2 group was significantly associated with the quinolone (CIP, NX, and NA) -resistant isolates (*P* < 0.05) (Fig. 1).

### Distribution of virulence genes in relation to antimicrobial susceptibility

Out of the 120 isolates, 39 isolates carried *pap* (32.5%), 26 carried *hlyA* (21.7%) and 21 isolates carried *cnf* (17.5%). Significant associations were found between the simultaneous presence of *cnf* and *pap* genes (*P* < 0.001) which corresponded to 14 isolates, between the presence of *pap* and *hlyA* (*P* = 0.009; *n* = 14 isolates), and between the *hlyA* and *cnf* (*P* = 0.017, *n* = 9 isolates).

To identify virulotypes of the isolates, profiles of the virulence genes were determined. A total of 56 (46.7%) isolates were found to harbor at least one of the three urogenes and the simultaneous presence of the three genes was detected in 7 isolates. The isolates exhibited 7 virulence profiles (Table 2). The most common profile was characterized by the presence of *pap* gene (*n* = 18 isolates) followed by the presence of *hlyA* (*n* = 10 isolates). The least distributed profile was the simultaneous presence of *hlyA* and *cnf*, which was detected in only two isolates. A comparison between B2 and non-B2 phylogroups showed a higher prevalence of three virulence genes in group B2, although it was not significant (*P* > 0.05).

**Table 2:** Virulotypes of 120 *Escherichia coli* isolated from urinary tract infection

Profile of virulence genes	Number of isolates (%)
*pap*	18 (15)
*hlyA*	10 (8.3)
*pap, hlyA*	7 (5.8)
*cnf, pap*	7 (5.8)
*pap, hlyA, cnf*	7 (5.8)
*cnf*	5 (4.2)
*cnf, hlyA*	2 (1.7)

To assess whether there is a relation between susceptibility and virulence, we evaluated the frequency of each virulence gene (*hlyA*, *cnf1*, and *pap*) in antibiotic-susceptible or -resistant isolates (Table 3).

**Table 3:** Distribution of virulence genes according to antibiotic susceptibility among 120 *Escherichia coli* isolated from urinary tract infection

Antibiotics	Prevalence of *pap* (%) in		Prevalence of *hlyA* (%) in		Prevalence of *cnf* (%) in	
	^b^**Sus. isolates**	**Res. isolates**	**Sus. isolates**	**Res. isolates**	**Sus. isolates**	**Res. isolates**
CIP (*n*^a^ = 40)	35	31.2	35	15*	22.5	15
TS (*n* = 34)	29.4	33.7	26.5	19.8	11.8	19.8
GM (*n* = 62)	32.3	32.8	17.7	25.9	16.1	19
Cax (*n* = 66)	33.3	31.5	28.8	13*	19.7	14.8
TE (*n* = 49)	30.6	33.8	28.6	16.9	18.4	16.9
NX (*n* = 74)	32.4	32.6	24.3	17.4	16.2	19.6
Caz (*n* = 77)	35.1	27.9	27.3	11.6*	22.1	9.3
NA (*n* = 53)	32.1	32.8	26.4	17.9	22.6	13.4
AK (*n* = 89)	29.2	41.9	22.5	19.4	13.5	29
Cpe (*n* = 55)	34.5	30.8	30.9	13.8*	23.6	12.3
Azt (*n* = 74)	33.8	30.4	25.7	15.2	20.3	13

*Abbreviation*: *CIP* Ciprofloxacin, *TS* Trimethoprim-sulfamethoxazole, *GM* Gentamicin, *Cax* Ceftriaxone, *TE* Tetracycline, *NX* Norfloxacin, *Caz* Ceftazidime, *NA* Nalidixic acid, *AK* Amikacin, *Cpe* Cefepime, *Azt* Aztreonam

^a^*n* = Number of susceptible isolates to the antibiotic

^b^*Sus.* Susceptible, *Res*. Resistant

^*^Comparison yielded a *P* value of < 0.05

In general, the *hlyA* gene was more prevalent in the susceptible isolates; the only exception was GM for which the resistant isolates showed a higher prevalence. The *pap* showed a more prevalence in the CIP- susceptible group and beta-lactam susceptible group (Cax, Caz, Cpe, and Azt) than in the -resistant group. The *cnf* was also more prevalent in the beta-lactam susceptible, TE-susceptible, and quinolone (NA, CIP)-susceptible isolates.

Subsequently, the possible significant differences between susceptibility and virulence were investigated (Table 3). The distribution of *cnf* and *pap* in relation to antimicrobial susceptibility was not significantly different (*P* > 0.05); however, an analysis on the *hlyA* revealed significant associations between the presence of *hlyA* and susceptibility to the tested cephalosporins (Cax, Caz, and Cpe), and CIP (*P* < 0.05). The most significant difference was emerged between CIP-susceptible and -resistant isolates (*P* = 0.012): 35% of the CIP- susceptible strains possessed *hlyA*, whereas 15% of the resistant isolates harbored *hlyA*.

The *hlyA* showed a meaningful distribution between the susceptible and resistant groups of cephalosporins (Cax, Caz, and Cpe) and CIP. The effect of sub-MIC Cax and CIP, two of the most common antibiotics used to treat UTI, was examined on *hlyA* expression by RT-PCR. For expression assays, seven isolates were randomly selected from the *hlyA*-positive isolates with susceptibility to both CIP and Cax. The sample size was determined based on the type of study. A similar sample size has been used by several similar studies to ours [23–26].

### Determination of MIC

The MICs of antibiotics (CIP and Cax) were determined for the seven UPEC isolates by the serial dilution method according to the CLSI criteria [17].

The CIP MIC values were as follows: 0.03 μg/mL for four isolates and 0.016 μg/mL for three isolates. In addition, the MIC values of Cax were: 0.12 μg/mL for four isolates, 0.06 μg/mL for two isolates, and 0.25 μg/mL for one isolate (Table 4).

**Table 4:** Isolates used for expression assay and their virulence profile, phylogroup, and real time PCR analysis

**Isolates**	**MIC (µg/mL)**		**Virulence profile**	**Phylogroup**	^a^**Fold change for Cax (decreased expression level)**	^**a**^**Fold change for CIP (decreased expression level)**
	**Cax**	**CIP**				
U41	0.06	0.03	*cnf-pap-hlyA*	B2	0.05 (20)	0.22 (4.54)
U51	0.12	0.03	*cnf-hlyA*	B2	0.17 (5.89)	0.16 (6.25)
U91	0.06	0.016	*hlyA*	F	0.3 (3.33)	0.67 (1.49)
U66	0.25	0.016	*cnf-pap-hlyA*	B2	0.4 (2.5)	0.95 (1.05)
U92	0.12	0.016	*cnf-pap-hlyA*	B2	0.02 (50)	0.12 (8.33)
U47	0.12	0.03	*hlyA-pap*	E	0.97 (1.03)	0.53 (1.89)
U56	0.12	0.03	*hlyA-pap*	D	0.06 (16.67)	0.14 (7.14)

^a^The reported data are fold changes in expression of *hlyA* after exposure to 0.25 MIC ceftriaxone (Cax) and ciprofloxacin (CIP)

Significant changes were seen when compared with the control (no antibiotics) (*P* < 0.05)

### Results of RT-PCR

The response of isolates to 0.25 MIC of CIP and Cax (i.e. 0.0075 or 0.004 μg/mL for CIP and 0.015, 0.0625 or 0.03 μg/mL for Cax) in regard to the *hlyA* expression has been quantified by monitoring the expression in the cultures of the seven isolates grown with and without antibiotics with 16S rRNA as a normalizer gene.

As shown in Table 4, the fold changes were from 0.02 to 0.97 in the presence of Cax and from 0.12 to 0.95 in the presence of CIP, when compared to the control (no antibiotics) by the paired t test (*P* < 0.05). Exposure to 0.25 MIC of both antibiotics led to decreased *hlyA* mRNA levels by 1.03 fold to 50.00 fold in the presence of Cax and by 1.05 fold to 8.33 fold in the presence of CIP, depending on the isolates. The exposure to sub-MIC Cax decreased the *hlyA* expression to a greater extent than treatment with sub-MIC CIP. The results obtained for the seven isolates are summarized in Table 4.

### Determination of hemolytic activity

**Fig. 2:**
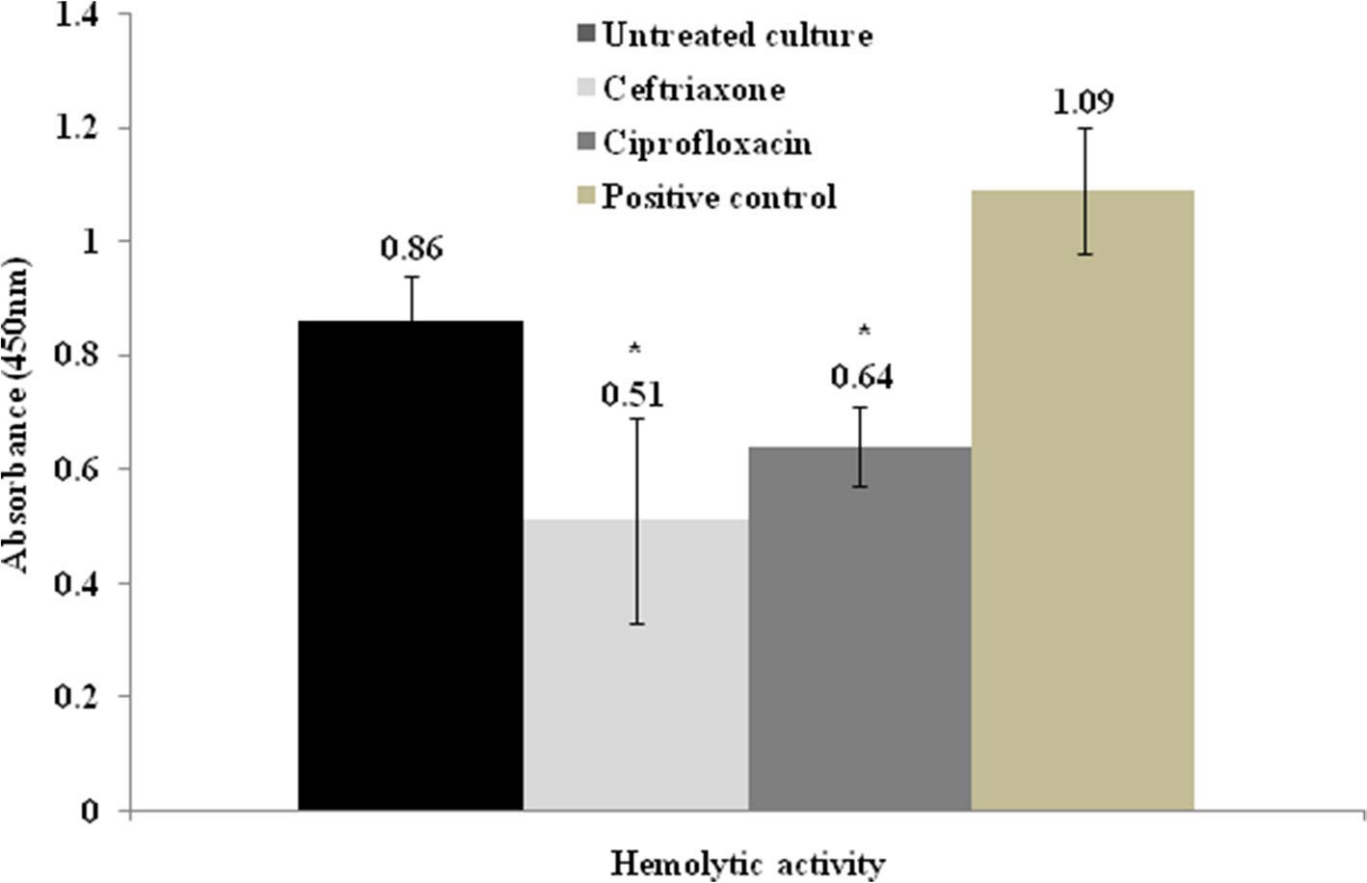
Effect of ciprofloxacin and ceftriaxone on the hemolytic activities of uropathogenic *Escherichia coli* isolates. Cultures were grown in the absence (untreated) or presence of 0.25 sub-inhibitory concentrations of antibiotics. Deionized water was used as a positive control. Values represent the mean ± standard deviation of three independent assays.* indicates *P* < 0.05 when compared to the untreated (antibiotic-free) culture

Supernatants of the seven *hlyA*-positive isolates co-cultured without or with 0.25 MIC of Cax and CIP were investigated using the hemolysis of sheep RBCs. The results were expressed as the mean and standard deviation (SD) of three experiments. For both antibiotics, the isolates showed a significant decrease in hemolytic activity (*P* < 0.05) in the presence of sub-inhibitory concentrations when compared to the antibiotic-free group (Fig. 2). The average A450 of the Cax-treated erythrocyte samples was 0.51 ± SD 0.18 and of the CIP-treated samples was 0.64 ± SD 0.07. The drug-free culture supernatants were served as the 100% hemolysis control and relative percentage hemolysis values of drug group were calculated by comparison to this value. When the isolates were cultured with sub-inhibitory concentrations of Cax, they showed almost half (50.7%, *P* < 0.05) of the hemolytic activity of the isolates grown in the untreated medium. The hemolysis value of the CIP-treated samples was 69.9% (*P* < 0.05) compared to the untreated cultures, which indicates that the hemolysis inhibition in the Cax-treated samples was greater than that in the CIP-treated samples. In addition, the presence of *hlyE* gene was investigated in the seven *hlyA*-positive isolates and no isolates carried the *hlyE*.

### Effect of antibiotics on growth rate

To confirm that the CIP and Cax-promoted decrease in the *hlyA* expression was not an effect of decreased growth rate, we analyzed the growth rates of the seven *hlyA*-positive isolates in the presence of 0.25 MIC of CIP and Cax in the growth medium.

**Fig. 3:**
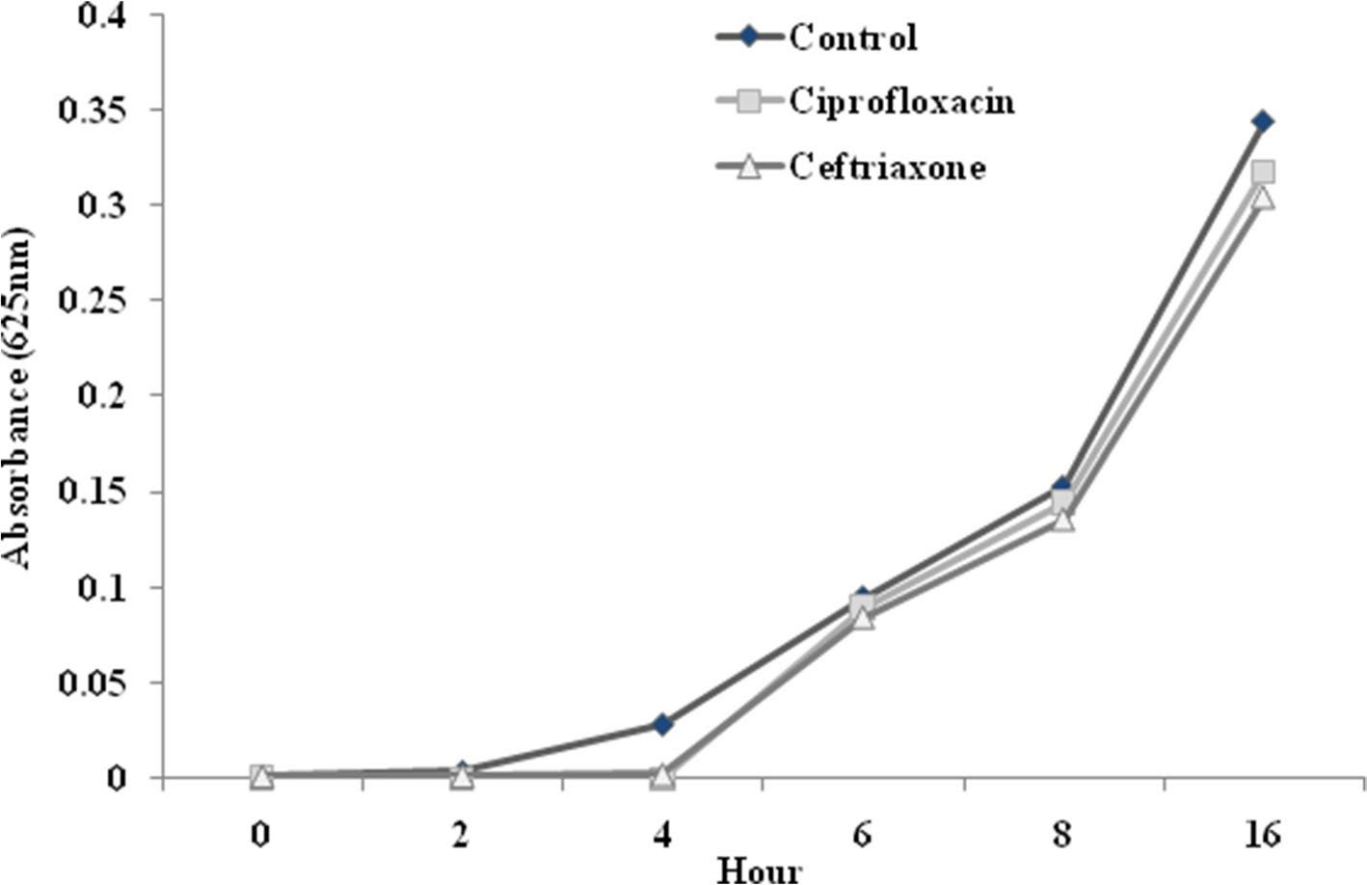
Growth rate analyses of uropathogenic *Escherichia coli* isolates. Isolates were challenged with or without 0.25 MIC of ceftriaxone and ciprofloxacin

Overall, during 4 h after the addition of antibiotics, the isolates displayed slower growth, compared to those grown in the antibiotic-free MHB. However, after 4 h, the increase in optical density of isolates was not affected by the presence of antibiotics in the medium, being at least equivalent to that in the antibiotic -free MHB (Fig. 3). Therefore, the differences between the growth rates of isolates in the antibiotic-free MHB (control) and in the supplemented MHB were not significant (*P* > 0.05), indicating that the decrease in *hlyA* expression was not an indirect effect of decreased growth rate.

## Discussion

Antibiotics, especially at sub-inhibitory concentrations, may act as signaling molecules aside from their antibacterial effect. Of particular concern is the possibility for antibiotics to increase expression of virulence genes [1].

Fluoroquinolones and beta-lactams are the two major groups for antibiotic therapy of UTI. In our study, the quinolone -resistant isolates were significantly associated with phylogroup B2. Piatti et al., also found that CIP-resistance was associated with significant shifts in the phylogenetic distribution among urinary *E. coli* isolates and mutations conferring quinolone resistance may require a particular genetic background [9]**.**

The dissemination of resistance genes is associated with mobile elements that may also carry virulence determinants. However, the interplay between resistance and virulence is complex and poorly understood [27]. For instance, a previous study demonstrated that resistance of *E. coli* strains to quinolones and TE, but not to ampicillin, amoxicillin-clavulanic acid, and GM, was correlated with the presence of fewer virulence factors [28]. While, another study showed a significant correlation between the presence of virulence factors and resistance to AK, ampicillin, and TS [29]. Houdouin et al., showed that ampicillin and TS susceptibility status did not influence the prevalence of virulence factors [30]; in contrast to Moreno et al., who showed that *E. coli* isolates resistant to quinolones and TS were associated with reductions in the virulence traits [31]. These findings emphasize the complexity of association between antibacterial resistance and virulence in *E. coli*. We observed a significant association between the presence of *hlyA* and susceptibility to Cax and CIP; therefore, we focused on whether these antibiotics have any effect on the *hlyA* expression. Adamus-Bialek et al., showed the loss of urovirulence genes in CIP- resistant derivatives of *E. coli* strains [1]. Soto et al., also found a loss of the PAI containing the *hly* and *cnf1* just after the passage in the presence of sub-MIC CIP. According to their results, the strains might have lost their PAI in exchange for resistance [2]; however, the finding that CIP-less susceptible mutants derived from hemolytic-susceptible isolates still produced hemolysis suggested otherwise [28]. Our study showed that expression of hemolysin was down-regulated at the transcriptional level. This finding may explain the phenomena observed in the previous studies [2, 28] which found that after quinolone exposure, some strains showed decreased hemolytic activities or had lost the capacity to produce hemolysis but they had not lost the *hly* gene. The MICs of CIP and Cax remained below 1 µg/mL, which indicates that these antibiotics may influence virulence without selecting resistant populations.

Some sources indicate that DNA repair mechanisms are related to the modulation of virulence by antibiotics [1, 2]. Fluoroquinolones and beta-lactams, both initiate the SOS response at sub-inhibitory concentrations. The SOS response is activated, as a result of DNA damage. Upon DNA damage*,* LexA, a transcription factor in the SOS system, can bind to a large number of promoters, leading to modulation of virulence expression [32]. Furthermore, presence of quinolones in the medium may lead to the mutational changes in *gyrA* and it is possible that altered DNA supercoiling in these mutants may affect the expression of genes involved in the hemolysis production [28]. The existence of mutations in the components of the hemolysin operon is also possible [2]. Experiments with *S. aureus* showed that the induction of hemolysin expression by beta-lactams depends on a specific interaction of the agents with PBPs. As a consequence, such an interaction may trigger the regulatory network that controls virulence determinants. It has been shown that a group of genes called the cell wall stress stimulon is induced upon treatment with the cell wall-active antibiotics in *S. aureus*, possibly resulting in the modulation of virulence. The details of the process remained unknown [33]. Although the impact of some antibiotics on virulence modification in *E. coli* was not evaluated, data obtained using antibiotics within a similar class or with a similar mode of action can help predict the potential effects of unstudied agents [32].

Previous studies also described the effects of antibiotics below the MIC on bacterial cell functions, including alterations of virulence properties. For instance, Goneau et al. [10] described an *in vivo* study by which mice receiving sub-inhibitory CIP treatment were more susceptible to severe urinary infections and frequent recurrences by *E. coli*. In *S. aureus*, exposure to sub-MICs of antimicrobial agents led to an increased expression of fibronectin-binding proteins [34] and alpha-toxin by fluoroquinolones [35], an inhibition of Panton–Valentine leukocidin by clindamycin and linezolid [23], and an induction of hemolytic activity by beta-lactams [35].

Studies have shown that hemolytic ability of UPEC is linked to the production of HlyA [7, 8]. We couldn’t find the *hlyE* hemolysin in our isolates; therefore, in vitro hemolysis was used as a test for production assessment of HlyA. The contribution of antibiotic-based alteration of hemolysin production to the pathogenesis of UPEC infections is difficult to evaluate. At high concentrations, HlyA kills eukaryotic cells by forming pores in the membranes, while at lower concentrations, the toxin can interfere with the host cell signaling pathways, cause apoptotic cell death and promote the exfoliation of epithelial cells [5]. Therefore, it can be speculated that it is not only the encoding of hemolysin but the regulation of its expression that affects the successful pathogenesis by a given UPEC strain.

## Conclusion

In summary, our study provides a description of the correlation between sub-lethal antibiotic treatment and virulence factor expression in UPEC isolates. Further studies, including animal models and clinical trials, may elucidate the mechanisms and effects of virulence modulation by antibiotics in UTI pathogenesis. In addition to classic antimicrobial therapy, a new strategy could be the integration of agents which suppress the products of infection-associated genes. This may lead to both health-related and economic benefits.

## Data Availability

All data generated or analyzed during this study are included in this article.

## Abbreviations

UTI: Urinary tract infection
MIC: Minimal inhibitory concentration
UPEC: Uropathogenic *Escherichia coli*
PAI: Pathogenicity island
PBP: Penicillin binding protein
CLSI: Clinical and laboratory standards institute
TBE: Tris–borate EDTA
CAMHB: Cation adjusted Mueller–Hinton broth
CFU: Colony forming unit
RT-PCR: Real time-polymerase chain reaction
BP: Base pairs
RBC: Red blood cell
Cax: Ceftriaxone
Caz: Ceftazidime
Imp: Imipenem
Azt: Aztreonam
Cpe: Cefepime
Cfx: Cefoxitin
CIP: Ciprofloxacin
NX: Norfloxacin
NA: Nalidixic acid
GM: Gentamicin
AK: Amikacin
NI: Nitrofurantoin
TE: Tetracycline
TS: Trimethoprim-sulfamethoxazole
